# Responsibility for the Environmental Impact of Data-Intensive Research: An Exploration of UK Health Researchers

**DOI:** 10.1007/s11948-024-00495-z

**Published:** 2024-07-25

**Authors:** Gabrielle Samuel

**Affiliations:** Department of Global Health and Social Medicine, King’s College London, Strand, London, UK

**Keywords:** Environmental sustainability, Environmental impacts, Research, Health research, Responsibility, Responsibilisation, Neo-liberalism, Regulation, Data

## Abstract

Concerns about research’s environmental impacts have been articulated in the research arena, but questions remain about what types of role responsibilities are appropriate to place on researchers, if any. The research question of this paper is: what are the views of UK health researchers who use data-intensive methods on their responsibilities to consider the environmental impacts of their research? Twenty-six interviews were conducted with UK health researchers using data-intensive methods. Participants expressed a desire to take responsibility for the environmental impacts of their research, however, they were unable to consolidate this because there were often obstacles that prevented them from taking such role responsibilities. They suggested strategies to address this, predominantly related to the need for regulation to monitor their own behaviour. This paper discusses the implications of adopting such a regulatory approach as a mechanism to promote researchers’ role responsibilities using a neo-liberal critique.

## Introduction

It is well-established that climate change is a consequence of human-caused greenhouse gas emissions and that it is important that as a society we reduce our fossil fuel energy consumption as an attempt to mitigate these changes. Researchers working in laboratories generate more greenhouse gas emissions than the average person (Greever et al., 2020; Knödlseder et al., 2022). In a 2008 publication, the United States Environmental Protection Agency quoted wet research laboratories that use chemicals, reagents and biological matter as typically using five to ten (and up to 100) times more energy per area than office buildings (EPA, 2008). Other environmental impacts include the reagents (chemicals) and materials (plastics and so forth) used in laboratories. Data-intensive research that takes place in ‘dry’ laboratories (computational, physics, and engineering laboratories that use data analytics, artificial intelligence methods, etc.) is also associated with environmental impacts related to the manufacture, use, and disposal of digital technologies.

Concerns about the environmental impacts of research have been articulated in the research arena (Gormally et al., 2019; Kaplowitz et al., 2012; Wright et al., 2008) and many sustainability initiatives are proliferating, driven by individual researchers, research bodies, as well as research organisations (Concordat, 2024; Dobbelaere et al., 2022). Nevertheless, little scholarly work has attended to the relationship between addressing, on the one hand, the environmental impacts of research, and on the other hand, individual researchers’ responsibilities. This is surprising given the large body of literature that explores the various responsibilities that are/should be attributed to a researcher’s role more generally [for example, see (Douglas, 2003)]. Questions remain about what types of responsibilities are appropriate to place on individuals as researchers and what responsibility might look like in practice.

This paper situates itself within the broader literature that follows the definition of role responsibility as that which arises from the particular professional role of researchers in society (Douglas, 2003). It starts from the assumption that empirically understanding how researchers view their role responsibilities is an important first step in considering what types of responsibility are appropriate to place on researchers. Specifically, the paper explores how, if at all, individual researchers perceive their responsibilities towards the adverse environmental impacts of their research as part of their role, focusing on UK data-intensive health research. Data-intensive research involves the collection and/or analysis of vast datasets with powerful data analytics and/or artificial intelligence (AI) algorithms, and the field was chosen for analysis because it is part of a broader study that is exploring the environmental impacts associated with data-intensive health research. The research question was: what are the views of UK health researchers who use data-intensive methods on their responsibilities to consider the environmental impacts of their research? Twenty-six interviews were conducted with UK health researchers who use data-intensive methods. Empirical findings demonstrate how, even when researchers perceived a role responsibility to consider environmental issues, difficulties emerged with ascribing such responsibility in practice. Researchers called for regulation of their individual behaviour. In the analysis, these findings are situated within a broader neo-liberal critique of agendas that focus on regulating individual behaviour.

## Researchers’ Role Responsibilities

There are two bases for moral responsibilities in modern life: *general* responsibilities we hold as humans and *role* responsibilities that arise from our particular positions in society (Douglas, 2003). Many scholars have explored *researcher role* responsibilities in detail. Some have argued that a researcher’s role in knowledge production provides special status that trumps some general responsibilities because the knowledge that is produced is perceived to be so valuable to society (for an overview, see (Douglas, 2003)). However, most argue that researchers’ roles (also) add additional responsibilities because they have unique access to knowledge that can have societal impacts that only researchers are able to consider given their expertise. Additional responsibilities have also been argued to arise when research is funded (directly or indirectly) by the state and more generally, as Bird (2014) states, research is ‘*carried out in the name of society as an expression and reflection of the society's needs, interests, priorities and expected impacts*’ (p. 170) [for a much more detailed review and discussion, see Douglas, 2003; Mitcham, 2003; Wäscher et al., 2020)]. Researchers’ role responsibilities can be categorised either by *how* research is conducted, that is, those associated with professional conduct and integrity, or those related to the potential *consequences* of their research on society (Mitcham, 2003).

Considering the potential *consequences* of research means being responsible for thinking about the potential societal harms that could come from their research and mitigating them as much as possible. Empirical research suggests scientists take these responsibilities seriously, taking steps to consider them during their research (Davis, 2012; Glerup et al., 2017; Wäscher et al., 2020). They are also governed through initiatives such as responsible research and innovation (Li & Cornelis, 2020; Owen, 2014; Stilgoe et al., 2013).

At the same time, resonating with literature in the philosophy of science, scholars question how individual researchers can be responsible for unknown outcomes of their research– outcomes for which they will have little control over–and how they can take individual responsibility for any outcomes of their research field more broadly (Collingridge, 1980; Davis, 2012; Wolpe, 2006). Other scholars, emphasise that these responsibilities can be enacted in a multitude of ways, including through collectivising and using divisions of labour to facilitate responsible research to make it more ‘do-able’ (McCarthy & Kelty, 2010; Politi & Grinbaum, 2020; Spruit et al., 2016).

While this debate continues, this paper aims to contribute to this literature by providing empirical information about researchers’ role responsibilities as they pertain to the adverse environmental impacts associated with their practices.

### Collective Responsibilities and Responsibilisation of the Individual

While much research has explored researchers’ role responsibilities associated with the *consequences* of research, this scholarship has paid little attention to how these responsibilities relate to addressing the adverse environmental consequences of research endeavours. This is surprising because there is a wide-spread environmental ethics literature that questions the moral responsibilities each of us should hold as humans when considering environmental harms (Gardiner, 2006). Much of the debate in this literature, which has primarily been particularly focused on climate change, has paid attention to questions of individual versus collective (individual plus institutions, states, and international organisations) responsibility. The latter has gained the most traction (Galvin & Harris, 2014; Jamieson, 2015; Schinkel, 2011; Young, 2011). These discourses have focused on the importance of individual behaviour change through a ‘responsibilisation of the individual’ (Butler, 2010; Rose, 1999), while recognising that certain individuals and institutions are more responsible than others because they have contributed more to environmental harms; because they have more resources to instigate change; or because the alternative premise (considering all individuals, organisations, and societies equally responsible) is perceived to be unjust (Fahlquist, 2009; Galvin & Harris, 2014; Vanderheiden, 2011). This ‘responsibilisation’ of the individual discourse now permeates much of societies’ response to climate change, which stresses the need for collective action and for all individuals and institutions to take responsibly for their own climate change contributions (Butler, 2010; Vanderheiden, 2011). In this study we see some of these narratives emerging in the empirical data.

### Environmental Impacts of Dry Lab Research

Environmental harms associated with data-intensive health research are related to the digital sector upon which this research relies. The digital sector includes data centres where data is stored and processed; digital devices, such as computers, laptops, phones, robots etc.; and the network of cables and infrastructures that connect them. The sector is estimated to account for between 2.1 and 3.9% of global greenhouse gas emissions (Freitag et al., 2021). In the UK, data centres account for about 2.5% of electricity consumption (NationalgridESO, 2022), 50% of which is associated with compute power with most of the remainder required to cool servers (computers) that become hot when used (Jones, 2018).^1^ The energy consumption of high performance computing (HPC; computing required for powerful data analytics such as for training self-learning (artificial intelligence) algorithms) has been assessed in some instances. One study reported that training a single natural language model (NLP; type of self-learning algorithm) can generate greenhouse gas emissions equivalent to the total lifetime carbon footprint of five cars (Strubell et al., 2019). In an example from the health sector, the energy required to conduct a genome-wide association study for 1000 traits has been reported to be equivalent to the production of 17.3 tonnes of carbon dioxide (Grealey et al., 2022).

A range of carbon calculators have been developed to allow researchers using data-intensive approaches to calculate the carbon equivalent emissions associated with their computing use (for example, see www.green-algorithms.org), though these exclude emissions associated with both the manufacture of the digital technologies and those associated with the individuals and organisations who will eventually use these algorithms. How much these exclusions matter will depend on algorithm uptake (how much society ends up using the algorithms) and the digital device upon which the algorithm is trained (how old it is, whether it is a new machine or a repurposed machine etc.).

Beyond carbon emissions, the digital sector has other environmental impacts. These include the need for minerals to manufacture technological components and the electronic waste (e-waste) associated with digital hardware disposal. While these impacts have attracted much less attention, in many ways these environmental impacts are most crucial to address. The detrimental health impacts associated with unsustainable mineral extraction and e-waste have been well-documented (Caravanos et al., 2013; World Health Organisation, 2021a). In terms of the latter, the digital technology sector contributes to the 50 million metric tonnes of e-waste produced every year (World Health Organisation, 2021b), which contains a range of hazardous materials (Mmereki et al., 2016, Rautela et al., 2021). Most of this e-waste is dumped on landfills in low and middle income countries (Forti et al., 2020). Many communities make a living through unregulated and informal e-waste recycling methods (e.g., open burning, incineration, acid stripping of metals, and acid baths) which generate hazardous by-products that have been identified at extremely elevated levels in those living nearby (Dai et al., 2020, Ngo et al., 2021, Singh et al., 2021). While steps are being taken to reduce the amount of e-waste through concepts of circular economy and the re-purposing of computers,^2^ e-waste levels continue to grow.^3^

## Methods

### Methodological Approach

This study is an exploratory, qualitative interview study. It does not aim to test a hypothesis nor to develop generalisable findings but rather to explore emergent themes in the data collected. As such, sampling for the interviews was not conducted to obtain a representative sample but rather was collected purposively to select potential interviewees based on a specific set of attributes–in this case those who research health-related topics using driven-intensive methodologies. This means that there are biases in the sample, not only due to the sampling method, but also because those who agree to take part in the study are likely to have more interest in the study topic (and those with less interest will not respond to invitations to participate). While this limitation would be problematic if the aim was to test a hypothesis, it is not the case here because the aim was to generate themes. These themes will need to be tested to determine how representative the findings are for broader demographics.

### Recruitment

Potential participants were identified via a number of routes: (a) a list of publicly accessible successful applications to access the UK Biobank resource for which principle investigator researchers were based in the UK, had publicly accessible contact details, and whose profile related to the use of data-intensive research methods (UK Biobank is population biobank in the UK that holds samples and associated data on half a million citizens); (b) a publicly accessible list of individuals involved in Genomics England GECIP (academic research partnership) groups whose profile related to the use of data-intensive research methods (Genomics England collected genomic sequences from 100,000 patients with a rare disease or cancer for research and clinical use); (c) checking publications from various bioinformatics journals for UK-based authors working in data-intensive research including, for example, *Biodata and Mining*, and the *Journal of Biomedical Informatics*; (d) searching Web of Science using keywords associated with digital health research associated with biosensing ((“mobile sensing”; (“wearables” and “health”); (“biosensors” and “health and data”); (“digital phenotyping”); (e) various web searches for data-intensive health initiative at various public and private institutions and organisations; and (f) snowballing. From this, a list of 145 relevant researchers were invited to participate. Following email invitations, 26 researchers agreed to participate.

### Demographics

The sample comprised mainly male participants (n = 21/26; in line with heavy bias in the field (Leavy, 2018)) and included nine professors; 13 research associates, fellows, lectures, or senior lecturers; one postgraduate (PhD) student; two employees of a small-to-medium (SME) company; and one health research data manager. Interviewees represented 14 different institutions and a number of disciplines and/or positions: clinical research (n = 6), engineering (including AI; n = 6), public health and/or epidemiology (n = 6), data science/bioinformatics (n = 4), health services research (n = 2), and data manager/curators (n = 2).

### Data Collection and Analysis

Interviews were conducted online or via phone between January and March 2022, were digitally audio-recorded (bar one, which was returned in written format), and lasted 25–65 min (n = 18 were over 40 min). The first interview was a pilot to test the interview schedule and minor changes were made following this interview. Interviews explored: participants’ background, use of data-intensive methods, and the type and quantity of data and methodologies used; participants’ understanding and views on issues associated with the environmental sustainability of data-intensive approaches, whether these were perceived as relevant to their own research, and if so, how they incorporated them (if at all) into their day-to-day decision-making; knowledge of relevant guidelines, and data storage locations and energy requirements; and considerations of responsibilities associated with sustainability considerations. The interview schedule is in the appendix.

Data saturation was reached during data collection, with no new topics or themes emerging during the final interviews. Analysis was inductive via two inter-linked rounds: broad coding (memo-making and scanning interview transcripts), and detailed coding using NVivo software (Strauss, 1987). Coding was carried out via constant comparison, which was continual, rigorous, and allowed for developing themes. No distinctions between disciplines/positions or seniority position were evident, perhaps because interviewees were self-selected and likely all were interested in sustainability issues. Limitations include the low representation of women and other demographic criteria. Furthermore, the sample is UK-based and the findings may differ if the study is repeated in other countries.

### Ethics Approval

The study received ethics clearance from King’s College Research Ethics Committee (MRM-21/22-26,574).

## Findings

Nearly (but not) all researchers perceived themselves as having a role responsibility to address the environmental impacts associated with their research. At the same time, they emphasised how they had thought little about this and had little understanding of how to enact any such responsibility. Furthermore, from what they did know–that they should be addressing energy and resource use associated with their methodological approaches–they struggled to understand how they could fulfil this responsibility because institutional arrangements meant they had little control over managing their data storage and analysis infrastructures. Therefore, and to differing degrees, participants deferred these responsibilities to their institutions, with most perceiving regulation of their own behaviour as the best mechanism to limit their energy and resource use.

### Environmental Impacts: Rejecting Claims of Researcher Responsibility

All participants expressed some general responsibility regarding environmental impacts of their endeavours. A minority of these participants did not perceive this responsibility to extend to their role as a researcher, which they viewed as superseding their general responsibilities–at least when considering energy consumption (n = 5). There were two main reasons for this. First, because this energy consumption was perceived as unfortunate but justified because of the innovativeness of their work.^4^ This was particularly the case for participants who trained machine learning (ML) algorithms. This was a field, explained interviewees, that was still developing both technically and conceptually and therefore required high energy exploratory work. In this work, interviewees stressed the need to facilitate research that involved training algorithms on datasets that have as many variables as possible (the more variables in the dataset, the higher the energy requirements to train an algorithm, and the lower chance of success, which then requires more training rounds). Interviewee 15 remarked:

increasingly the frequency of applications [to the data repository]…are just saying, “oh, can I just have 20,000 variables because I'm going to just release either an algorithm or some self-educating process on those data”. We just want to let it run…and that's the type of science that we want to facilitate.

As with any technological development, these interviewees described, the discipline would eventually streamline and become more efficient, but for now, using a lot of energy to conduct their research was acceptable. As such, while these participants perceived themselves to have a general responsibility to consider environmental harms, this responsibility was not attached to their specific research because high energy processes were intrinsic to their research field and to future discovery and new innovations.

Second, interviewees appealed to worse problems by viewing their energy consumption as relatively minor compared to other data-intensive sectors and/or research approaches. One interviewee compared their research to manufacturing and Bitcoin, which they viewed as much more energy intensive: ‘it’s [my research’s environmental impact is] too little compared with the manufacturing side and with Bitcoin’ (interviewee 11). While these researchers perceived value in considering the environmental impacts associated with data-intensive approaches, they viewed the energy consumption of their own actions as too small to worry about and so they rejected the more specific individual researcher role responsibility to consider this energy use. Furthermore, they perceived that even if their energy usage was relatively high, minor changes in their own actions were unlikely to be impactful when considering the overall changes needed to address issues of climate change and other environmental concerns. As some of these and other interviewees explained, many health researchers use ‘off-the-shelf’^5^ machine learning algorithms. They explained that these algorithms, which have already been trained by others and made available, consume low levels of energy because they do not require energy to train the algorithms from scratch but only use energy associated with the modification of the algorithm for a specific purpose. Participants did not connect this low energy use to the high energy likely required to initially train the algorithms, nor to other environmental impacts that may come from the use of these algorithms, i.e., those involved in the manufacture and disposal of digital technologies, though it was unclear whether this was because of a perceived lack of responsibility to these more upstream/downstream processes or because they had limited awareness of the issue because nearly all participants were considering these issues for the first time (‘it’s the first time I think of this’ (interviewee 11)).

### Environmental Impacts: Accepting Claims of Researcher Responsibility

In contrast to the views of participants described above, most interviewees felt a sense of responsibility to consider the environmental impacts of their work entwined with their role as a researcher. Those interviewees who had already considered this prior to the interview spoke about the different ways in which they already tried to enact these responsibilities. For example, interviewee 13 described how they made their research outputs open access to ensure the algorithms they had trained were used as much as possible to offset the energy and resource required to train the algorithms: ‘when you train an algorithm, you kind of burn this cost along the way. I think releasing the trained model up to that point is very helpful…[because other researchers] don't have to do all the retraining themselves, there's an existing thing…to just build on that’. In another example, interviewee 10 described how they attempted to reduce the environmental impacts of their own research by optimising their algorithmic code to run faster (and therefore use less energy), which they perceived as best engineering practice: ‘being a computer engineer by training…you try to optimise your code to run fast and run with minimal resources’. In a final example, interviewee 23 described how they were teaching students to only run (train) their algorithms if the students had ensured they had checked over their work (the algorithm’s code) as well as checked the dataset they were using to train the algorithm was properly cleaned (processed) and ready: ‘particularly for some of the Bayesian methods, they run for a very long time. I’ve said [to students] “don’t set those running unless you’re sure that the data is ready”’.

Despite this desire to take on a role responsibility, nearly all interviewees framed responsibilities associated with the adverse environmental impacts of their research as often being inconsistent with their daily experiences. That is, in their daily experiences there was little opportunity for them to take responsibility for the environmental impacts of their research in terms of them having control to make any relevant decisions and/or changes.

### Struggling to Enact Responsibilities

Besides the examples provided above, nearly all participants did not know how to reduce the adverse environmental impacts of their research and were unaware of any useful benchmarks that could help them better understand their research’s environmental cost: *‘*without having a benchmark [it is hard to know what to do]—is me running a process the same as me driving to Tescos [UK supermarket]…?’ (interviewee 5). Furthermore, they were unsure how to advocate for change given that they were unaware of what beneficial in terms of making changes to decrease the environmental impacts of their research looked like: ‘I can’t really say to a high performance computing cluster, sorry,…could you just look into XYZ. I wouldn’t know…what they could do to be more sustainable’ (interviewee 3). Interviewee 21, who trained natural language programming (NLP) algorithms on electronic health records, explained how they had seen a comment on twitter about the environmental impacts of data-intensive approaches but was unsure how true it was and/or what to do about it:

I read somebody saying on Twitter, “are you aware that generating this text uses as much energy as sending a Saturn five rocket to the moon”. And I don’t know whether that’s actually true…I’m concerned about [it], but I’m not sure what to do about it.

Interviewees called for more awareness on these issues so they could give them consideration. Interviewee 25—a strong proponent of sustainable open science—described various bottom-up approaches adopted within their community that could be useful, including energising researchers, funding the topic, and running conferences.

Furthermore, and crucially, nearly all participants described how their data practices (where data was stored, processed, and where algorithms were run) were often managed by their institution, by a data curator, or by an external cloud provider. Interviewees therefore perceived themselves as having little agency or control to enact change*.* Some interviewees compared this lack of control to their ability to control their research-related travel*.* Other interviewees explained that because it was difficult to ‘see’ the environmental impacts of data-intensive research, it was equally difficult to consider these issues.

These constraints meant that participants viewed their capacity to fulfil their responsibilities as related to the opportunity to do so. Because this opportunity was often missing, interviewees perceived responsibility to lie at the institutional level.

### Collective Versus Institutional Responsibility

Interviewees attached different meanings to advocating institutional^6^ responsibility. For many, it was associated with collective responsibility. In this case, participants delegated only the obligation of understanding *how* to address the environmental impacts of their own work to their institutions. These interviewees envisaged a division of ‘responsibility labour’ [c.f. ethical labour (Politi & Grinbaum, 2020)] within the research community, with some individuals (within their own department, or more broadly in the institution) taking more responsibly for understanding how to address research’s environmental impacts and others being more responsible for changing their behaviours. The former were described as being in a ‘position to lead’:

we all have the same responsibility but some of us are in a better position to lead the effort and make it happen than others. You have to have the knowledge around this… it really is for the experts to work out what is best and then sell it to the rest of it (interviewee 1).

This division of labour meant interviewees wanted to be prompted to think in a certain way and needed to be formally ‘given the responsibility’ to do so: ‘I consider myself reasonably environmentally concerned…but running a project like this…the questions [are] not asked so, you’re not prompted to think about it’ (interviewee 25); ‘I don’t have a responsibility at the moment formally, nobody’s given me that’ (interviewee 24).

The division of responsibility worked differently for a lesser number of interviewees who were happy to defer *all* responsibilities to the institution (institutional responsibility; no collective responsibility): ‘I think someone definitely needs to think about it, say at the university level, where it’s more fundamental than individual researchers’ (interviewee 13). This was especially important to participants who perceived that researchers—including themselves—had other research-based priorities (technical, ethical, etc.) that needed to be addressed. Data management teams were considered appropriate to take on this role because of their long-time responsibility for managing data governance issues, even in pre-digital times (interviewee 26). Interviewee 21 described how they ‘go along with whatever the IT [information technology] department tells us to do’. In other instances, participants looked to curators of large databases (public and private)(interviewee 15). Though most prominently, participants pointed to research institutions and regulators (funding bodies, businesses, journal editors) to fulfil this role. In fact, some interviewees *assumed* that environmental factors were already being addressed at this level: ‘you have to assume the university, someone there is dealing with that side of things’ (interviewee 13). Finally, some interviewees believed that the causes of the environmental impacts of research were so endemic, that solutions should not be positioned solely within the research community but needed addressing at the level of government: ‘it needs to come from government’ (interviewee 23); ‘the whole infrastructure of the country needs to be adapted’ (interviewee 1).

### Institutional Responsibility: The Need for Regulation

For the majority of interviewees who called for collective responsibility, a small minority viewed their own responsibilities within this conception as something that should remain unmandated. For some of them, feedback on the amount of energy consumed as part of their research was sufficient: ‘you press enter to submit a job. There is absolutely no feedback…some kind of email that …said “do you realise your computation processes this week was the equivalent of heating 30 houses [would be useful]”’ (interviewee 23). Others described the need for research institutions to instigate incentives to change practice. For example, interviewees 12 and 14 explained how, in the current un(der) regulated environment no cost is attached to data collection, use, or processing, and so there was little incentive to change behaviour. Adding a cost could make researchers think more about whether a particular computer job (algorithm) was absolutely necessary to run: ‘compute power is really easy to use here because it’s free, so it makes us think less about whether we want to run something or not’ (interviewee 14).

The majority of participants, however, leant heavily towards the need to regulate researcher behaviour through mandatory governance. In essence, not only did these interviewees want their institutions and regulators to tell them what to do, they wanted them to mandate it. While these responses could be interpreted as a way to shift responsibility (and blame) away from themselves as individual researchers towards the need for more organisational and even national governance, analysis of the findings suggested that this was not what the interviewees meant. Rather, they emphasised the benefits of regulation–and in particular regulation that regulated themselves–and advocated a range of potential regulatory approaches that could be taken not only at the level of research institutions and business, but also at the level of publishing and funding. For example, interviewee 10 described one possible approach to regulation that involved compulsory reporting of computational times and energy consumption in peer reviewed articles: ‘in the past…it was almost a prerequisite that you publish computation times [in engineering journals]….and it wouldn’t be too difficult to integrate some of these aspects’ (interviewee 10). In another example, many interviewees viewed it important for funding bodies to impose requirements on themselves to forecast the potential environmental harms associated with any research proposals and to explain how they could take steps to mitigate them: ‘if that came from the funders I think that will be a huge motivation for people to start thinking about these issues’ (interviewee 3).

Interviewees emphasised that their research environment was already heavily regulated and so any such additional regulation could just be incorporated into this: ‘we’re all used to them, you know, there’s a huge bureaucracy around research and we generally comply with it’ (interviewee 6). In fact, interviewee 25 spoke about relying on such a system: ‘you kind of rely on the system of regulation, because so much else of what we do is regulated’.

## Discussion

Most interviewees believed researchers should have a role responsibility to consider the adverse environmental impacts of their research and they themselves wanted to take on this responsibility; in fact, several participants were actively thinking about how they could do this in practice or were already implementing these responsibilities in particular ways. At the same time, participants recognised the limitations of, and challenges to, any such role responsibility. There were no clear norms with which to enact this responsibility and so they had limited understanding of how to do this and had difficulty characterising it. Moreover, what they did understand about the issues seemed inconsistent with their day-to-day research endeavours because of broader institutional arrangements that meant decision-making about the environmental impacts of their research sat outside the scope of what they were able to change. This meant making their responsibilities ‘do-able’ was a practical problem. This was an issue because a key aspect of being responsible is having the capacity to act; if you do not have the ability to act, then it is difficult to be responsible–being responsible is being ‘response-able’–being able to respond (Johnson & Michaelis, 2013).

As participants attempted to grapple with these issues, it made sense to divide labour by deferring responsibility to their institutions. At the same time, most participants did not completely reject their responsibilities—as with researchers’ broader desires to consider wider social responsibilities associated with their role, they wanted to be part of the response to addressing the environmental harms associated with their research and viewed this as a collective endeavour. This desire to respond could also be because researchers were aligning themselves with broader societal discourses that frame the need to address environmental harms through collective action. In their view, such responsibilities could be achieved through institutions dispatching various regulations to researchers so that their behaviour could be monitored and regulated. In essence, they were calling for (and even wanted a reliance upon) responsibility to be institutionalised through what has been conceptualised as standardised disciplinary norms and institutional procedures (Gunzenhauser, 2013; Shore, 2017; Shore & Wright, 1999).

### Regulation as Part of the Neo-Liberal Agenda: Problematising this Approach

Regulation of individual behaviour through standardised disciplinary norms or compliance-based approaches can be conceptualised as an aspect of neo-liberalist agendas that dominate modern societies. Neo-liberalism is a type of governing structure that constructs problems in ways that renders them as problems that need to be dealt with by individuals. In this way they place responsibly onto individuals and require individuals to be accountable (‘responsibilisation’ of the individual). To achieve this aim, and to ensure that individuals’ behaviours align with the aims of the governing body, governing requires regulation of individual behaviour. This allows individuals to be ‘surveyed, measured against the “norm”, [and] trained to conform’ (Shaw, 2007, p. 319). Through regulatory mechanisms, the aim is to encourage individuals to adopt new norms and values that transform their conduct to ensure ‘behaviour is conducted according to appropriate standards’ (p. 319).

Few would argue that regulation towards reducing the adverse environmental impacts of data-intensive technologies is a bad thing. Such regulation can standardise practices. Furthermore, while any accountable system moralises actions into irresponsible and responsible (Butler, 2010), participants hinted that such a system was not perceived as a repressive force, but rather, as other scholars have suggested, a productive one that operates through the knowledges which create people as disciplined subjects, and as an organised practice through which they could be governed and through which they could govern themselves (Henderson, 2015). This system was viewed by participants to provide an approach to allow them to make the role responsibilities they perceived they had towards addressing the environmental impacts of their work ‘do-able’ and allow them to make a responsible contribution to collective action.

Nevertheless, there has been much critique of (neo-liberal) regulatory approaches that aim to govern individual behaviour. Drawing on these critiques, we can hypothesise several issues with adopting a regulatory approach to govern researchers’ role responsibilities associated with mitigating the environmental impacts of research.

### Regulating Individual Researchers: a Critique

Many scholars have critiqued regulatory approaches that aim to influence individual behaviour. Most prominently, these regulatory approaches are viewed as a way to off-load responsibility away from institutions towards individuals. This is because regulatory approaches require individuals, as rational agents, to remain compliant. Non-compliance is explained by a lack of individual self-control to normalise behaviour to regulatory mechanisms. Rather than a collective responsibly, then, regulatory approaches promote a ‘responsibilisation’ of the individual (Rose, 1999) in which responsibility is constructed in terms of following regulatory mechanisms, any divergence from which becomes about individual rather than collective blame.

Second, placing responsibility on individuals through such regulatory approaches has been argued to result in rule-based compliance rather than the development of a responsibility towards the rule’s rationale in the first place, i.e., tick-boxing rather than deep reflection. This is because compliance through regulation is based on the idea that individuals make decisions in a rational-centric way based on reason alone. This is problematic because, third, it fails to consider that responsibilities are behavioural and embodied and that ‘our actions, attitudes, and attachments take shape at not only the cognitive, but also the visceral, level’ through our emotions and feelings (Ellis, 2022, p. 5). This means that rule following does little to modify behaviour except for requiring compliance. As such, if we want to construct notions of responsibility there must be a realisation that decision-making is embodied through feelings and that alongside rules and regulations, there is also a need to alter affective environments in which habits are formed to nurture concerns about the environmental impacts of research (Ellis, 2022). This means we need to provide a space and/or context that allows researchers to reflect on the environmental impacts of their research and to be able to address this as they see appropriate within their roles. If we do not provide this space/context, researchers may just follow the rules so that, fourth, when aspects that need to be addressed are not included in the rules and regulations, they go unaddressed. For example, this type of regulation can lead to instances in which researchers perceive their responsibilities are over, once regulation is followed, as has been seen in other fields, such as research ethics regulation (Samuel et al., 2021). This is problematic because some aspects of addressing environmental harms may not be included in the regulation. For instance, while energy/resource savings could be supported through regulation of individual behaviour, these savings may be used to support additional research/consumption elsewhere if no additional regulation is put in place, thereby not reducing consumption overall. In another example, regulation of energy consumption pays little attention to how many computers researchers are buying, thereby missing important environmental harms related to resource extraction and e-waste.

Fifth, the instigation of rules and regulations has been argued to not allow for decisions to be tailored to specific circumstances because they enact centralised regulation in a rigid and standardised way. This lack of contextualisation means that following rules and/or regulations can lead to morally problematic outcomes (Dekker, 2020; Gunzenhauser, 2013; Lyle et al., 2022; Wäscher et al., 2020). For example, questions emerge about how such a rigid regulation could reflect on the *purpose* for which energy is used. Furthermore, questions around whether carbon emissions or energy should be regulated raise questions about inequities in terms of unequal access to renewable energy. Finally, the introduction of top-down rules may not be straightforwardly accepted by (all) researchers. Rules may be viewed as ‘yet another bureaucratic hurdle imposed in a top-down fashion’ (Politi & Grinbaum, 2020).

### Improving Regulatory Approaches

The above concerns have led some scholars to consider how to deliver better rules. Some call for flexible and permissive regulation so that they encourage attention to the nuances of specific contexts and cases, or for decisions to be tailored to specific circumstances (Lyle et al., 2022; Sorbie, 2020). Others highlight that rules can only function if investment and effort is made into understanding and interpreting their meaning–by both those following the rules and/or by those delivering them (Davis, 1999). The alternative–blind following or strict obedience–means rule following remains non-context specific or relevant to particular situations (Davis, 1999). Both options to a regulatory approach warrant further attention because while not without limitations, and while steps must be taken to ensure that rules do not end up placing responsibility solely on the individual, rules and regulations are key to any governance structure and can have positive effects. Examples in broader society include regulating the cost of plastic bags at shops in various jurisdictions, including the UK, which reduced the use of plastic bags and built public awareness around the issue (Thomas et al., 2019).

At the same time, this plastic bag tax did not necessarily shift broader consumer practices around environmental sustainability (Laranjo, 2021). Complimentary approaches that move away from the idea that role responsibility is *only* about bureaucratically following rules are also needed.

### Other Approaches to Discharging responsibilities: Beyond Regulating How Research is Conducted

This paper follows an approach to responsibility that best engages and motivates change (Jamieson, 2015). One approach to this, discussed in the introduction, views role responsibilities as relating to organising collectives, through which awareness building, advocacy, guideline development, and the use of/taking part in accreditation mechanisms can allow researchers to take on responsibility. We already see such collectives through bottom-up initiatives that promote environmentally sustainable research practices (Dobbelaere et al., 2022). Another approach is to view role responsibility at the level of the research community rather than at the individual level so that it is discharged and stabilised through funding streams and the creation of new institutes around matters of environmental concern. To implement this type of responsibility requires funding bodies and other research institutions to embrace responsibility through resourcing such initiatives and to do so in a way that steers the research agenda to focus on questions that relate to reducing environmental harms in (research and) society, and for researchers to take part in such research. In this scenario, rather than responsibility being focused (only) on *how* research is being conducted, responsibility is focused on *what* research is conducted. Role responsibility for researchers then moves away from only being about individual researcher role responsibility towards a responsibility that involves many research ecosystem actors and, as others have argued, responsibility is constructed as the development of research questions that align with environmental goals, making it a positive experience because it provides researchers with opportunities to follow these agendas in ways that are scientifically interesting to them, and in ways that require scientific creativeness to develop solutions using new and innovative approaches (McCarthy & Kelty, 2010). This does not negate the need for any regulation, but offers a complimentary approach.

## Conclusion

This study aimed to answer the research question: what are the views of UK health researchers who use data-intensive methods on their responsibilities to consider the environmental impacts of their research? Participants perceived researchers to have a role responsibility to consider the environmental impacts of their research, however in many cases, they perceived they had little control to affect change in their daily practices. They viewed regulation of their behaviour as one solution. This paper does not reject this approach but problematises it by drawing on neo-liberal critiques. Furthermore, it does not reject the claim that researchers have a role responsibility but argues that we need to think carefully about what it would mean to have a role responsibility as a researcher and whether this is better (should also be) discharged at the research ecosystem level. While this research study is only small, and while the findings are just exploratory, their implications raise questions that can contribute to these debates.

## Data Availability

Data is not available open access due to consent restrictions. De-identified transcripts for some participants may be available on request if participants provided consent.

## Footnotes

1 Servers are the computers that are used to store and process data. These become very hot when working and need to be cooled down to ensure they work properly. https://www.energy.gov/eere/iedo/energy-efficient-cooling-control-systems-data-centers.

2 For example, see https://circulardigitalhealth.eu/.

3 https://www.statista.com/topics/3409/electronic-waste-worldwide/#topicOverview.

4 This aligns to broader arguments that perceive researchers’ role responsibilities as not needing to align with their general responsibilities because of their special status in knowledge production.

5 Algorithms that have already been trained for use and developed into software.

6 Most interviewees were affiliated with Higher Education Institutions (HEIs) and so much of the discussion revolved around aspects of HEI governance.
